# Deformation of the Titanium Plate Stabilizing the Lateral Ankle Fracture Due to Its Overloading in Case of the Young, Obese Patient: Case Report Including the Biomechanical Analysis

**DOI:** 10.3390/diagnostics12061479

**Published:** 2022-06-16

**Authors:** Grzegorz Szczęsny, Mateusz Kopec, Tomasz Szolc, Zbigniew L. Kowalewski, Paweł Małdyk

**Affiliations:** 1 Department of Orthopaedic Surgery and Traumatology, Medical University, 4 Lindleya Street, 02-005 Warsaw, Poland; grzegorz.szczesny@wum.edu.pl (G.S.); pawel.maldyk@wum.edu.pl (P.M.); 2 Institute of Fundamental Technological Research, Polish Academy of Sciences, 5B Pawińskiego Street, 02-106 Warsaw, Poland; tszolc@ippt.pan.pl (T.S.); zkowalew@ippt.pan.pl (Z.L.K.)

**Keywords:** ankle injuries surgery, bone plate, obesity, postoperative complications, implant failure

## Abstract

The number of overweight and obese patients in developed countries is gradually increasing. It was reported that 1287 (64%) out of 2007 adults operated on in 2017 had a body mass index (BMI) greater than 25 kg/m^2^, and 26.4% even greater than 30, while the BMI of the most obese patient was as high as 57.6 kg/m^2^. Such distressing statistics raised an issue related to the inadequate durability of implants used for the fixation of bone fractures. Implants for the lower-extremity fractures may not be durable enough to fit the requirements of overweight and obese patients. This case report presents the history of a 23-year-old obese male with a BMI of 38.7, who bent the angularly stabile titanium plate stabilizing his broken lateral ankle and torn distal tibiofibular syndesmosis. Biomechanical analysis showed that the maximal static bending moment registered during one-leg standing was equal to 1.55 Nm. This value was circa one-third of the maximally admissible bending moment for this particular plate (5.34 Nm) that could be transmitted without its plastic deformation. Since dynamic forces exceed static ones several (3–12) times during typical activities, such as walking, climbing the stairs, running, and jumping, unpredictable forces may occur and increase the risk of loosening, bending, and even breaking implants. None of these situations should have occurred for the typical patient’s body mass of 75 kg, or even for the analyzed mass of the young patient (120 kg) who tried to avoid excessive loading during his daily routine. Subsequent implant bending and destabilization of the fracture shows that for the significantly high and still growing number of obese patients, a very strict physical regime should be recommended to prevent overabundant dynamic loads. On the other hand, the geometry of implants dedicated to these patients should be reconsidered.

## 1. Introduction

Destabilization of bone fractures due to loosening, breaking/angulation of implants, and even pull ups of their screws from the bone, represent a considerable group of postoperative complications [1]. They usually occur either due to material manufacturing defects or excessive overloads. In most cases, these overloads lead to permanent deformation of the implant resulting from dynamic, cyclic, and complex loading, which is often a combination of tension, compression, bending, and torsion [1]. Such types of overloads may originate from unfortunate and unpredictable secondary injuries. In many cases, however, they occurred as a consequence of non-compliance with postoperative recommendations, which may happen in the case of insubordinate patients, especially young males prone to risky, physical activities [2]. In recent literature devoted to the numerical analysis of implant mechanical behavior, the body mass of ca. 75 ± 5 kg is usually considered as average. Nevertheless, even the average mass greater than 5 kg [3], that corresponds to calculations for 80 kg patients, is not representative for almost half of the adult population treated in our department. The socioeconomic changes observed in North America and Europe in the second half of the 20th century improved the availability of cheap, energy-dense food [4,5]. The simultaneous reduction of physical activity provoked by mechanization of work places and transportation resulted in a rapid increase of body weight. Consequently, the percentage of the overweight and obese population rapidly increased. Historical data show that in 1913, i.e., before the 1st World War, the percentage of the obese population living in the Warsaw district did not exceed 2.3%. Furthermore, more than 90% of people were classified as being of the recommended, that is, “healthy”, weight. An average male body mass and height was 60 kg and 165 cm, respectively [6,7,8]. Thus, the calculated BMI was equal to 21.9 ± 1.9 kg/m^2^, which is remarkably different than that calculated for the modern man. During the last century, the BMI changed significantly, being reduced during both world wars and directly after them. Afterward, it constantly increased, with a sudden burst that started in the 1990s. This effect was observed not only in Poland, but throughout the whole world [9,10,11]. As the average human body weight significantly increased, higher mechanical loads needed to be carried by implants. It should be highlighted, that application of implants with inadequate strength for a patients weight ratio considerably greater than the regular one may lead to implant overloading, and its subsequent loosening and breaking [12]. Unfortunately, loading of the lower extremities does not result from walking only [13]. Climbing down the stairs, jumping down from even a slight altitude, and many other activities significantly increase acting forces as well. Moreover, walking on an irregular surface introduces additional forces that do not act exactly along the mechanical axis of the limb. In such cases, a generation of additional forces may lead to deformation of or even destruction of orthopedic implants. Among other situations during which overloads occur include uncontrolled falls from stairs, ladders, or tables, and even blindsiding of pedestrians. All these situations may produce forces and moments of forces (moment of bending and twisting) that act on the limb in flexion, adduction, and valgus, and may result in temporary loading of magnitudes exceeding those allowable by the implant. The patient’s age and sex should also be considered in the analysis of the potential causes of the implant’s damage [14]. Young males are prone to risky, physical activities and are more likely disobedient to precautions given by a physician [2]. Interestingly, the overloads in most cases remain unrecognized. It should be mentioned that humans possess quite a good ability to estimate weight and mechanical loads by the upper extremities. Unfortunately, the possibility to estimate loads by a leg is very poor, and people are unable to validate it with an accuracy higher than 50–100 N. Consequently, patients are not aware of loads that they generate during typical, everyday activities, unwittingly overloading implants used for fracture stabilization. As a matter of fact, such loads should not influence an implant’s deformation in the case of a patient with a regular body mass, i.e., of ca. 75 kg.

To demonstrate the effects of the above-mentioned observations in the current orthopedic surgery, in this paper, bending of the titanium plate stabilizing the lateral malleoli was analyzed for the case of a young obese male, who overloaded the limb during a walk. As a result, the prior position of the implant stabilizing the broken leg was changed due to its bending, which led to broadening of the ankle joint and its instability, risking implant breakage or loosening. Such material behavior led to the question of whether implants for the lower-extremity fractures are durable enough to fit the requirements of overweight and obese patients.

## 2. Materials and Methods

### 2.1. Patient and the History of Treatment

The 23-year-old male was operated on due to a fracture of the lateral ankle of the left limb, corresponding to a disruption of the distal tibiofibular syndesmosis (DTFS; Danis-Weber type C) [15]. The fracture was stabilized with an angularly stabile titanium plate, and syndesmosis was anastomosed with two titanium cortical screws (System 5.0, ChM, Lewickie, Poland). The dynamic examination revealed an intact deltoid ligament in the intraoperative X-ray. The postoperative wound healed properly, and the patient was discharged from the hospital with the limb immobilized in a plaster cast. This patient was allowed to walk using forearm crutches without weight bearing on the operated limb.

Nevertheless, an ambulatory control performed a few days later revealed an edematous calf, compression of the postoperative wound by the plaster cast, and swollen and reddened wound edges with an exudation. The patient reported a partial limb loading while walking. The laboratory venous blood tests showed a slightly elevated leukocyte count (11.9 × 109/L), and normal C-reactive protein (4.5 mg/L) and procalcitonin (0.04 μg/L) values. The patient denied a fever.

The plaster cast was removed, and the limb was immobilized in a long walker boot orthosis. The microbiologic culture of the fluid from a puncture of the thin reservoir-residing subcutaneous tissue under the postoperative scar, diagnosed using ultrasonography, revealed a scanty ingrowth of the *Staphylococcus epidermidis* colonies, that were not resistant to the antibiotics. Consequently, oral clindamycin (3 × 0.3 g) was prescribed for 10 days, resulting in a remission of the wound swelling and redness.

**Figure 1:**
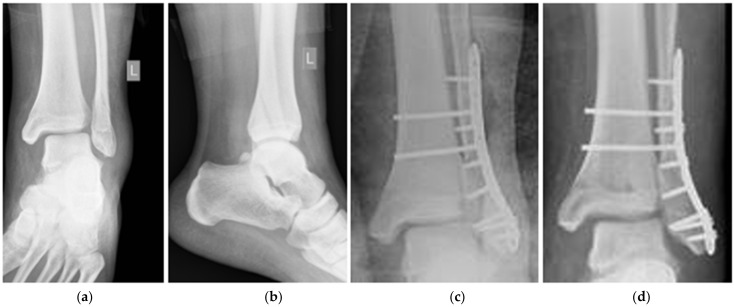
PA X-rays: (**a**) preoperative—front view, (**b**) preoperative—lateral view, (**c**) postoperative after stabilization, and (**d**) postoperative six weeks later.

The patient reported that due to psoriasis, he was locally treated with steroids. He also presented with obesity, i.e., body mass of 120 kg under a height of 176 cm (BMI of 38.7 kg/m^2^). The consecutive X-ray controls during the forthcoming three months revealed proceeding of a valgus bending of the plate, which stabilized the lateral malleolus corresponding to broadening of DTFS, a valgus dislocation of the trochlea of the talus, and stretching of the deltoid ligament, as shown in Figure 1. The patient reported a partial loading of the limb, while walking, despite a total prohibition of limb loading.

In the 12th postoperative week, the patient was re-operated on, and the plate was replaced with one of the same type, only longer (system 5.0, ChM, Lewickie, Poland). The DTFS was anastomosed by the use of three cortical titanium screws. The limb was immobilized in the plaster cast for six weeks and the patient was strictly forbidden to load the extremity. The consecutive X-ray controls showed a proceeding healing in the anatomical position. The intensive rehabilitation, that began directly after the plaster cast removal, enabled to restore a range of the motion and limb functionality to an efficiency comparable to that of the contralateral one. The fracture healed uneventfully, and the syndesmosis was efficient.

### 2.2. An Estimation of the Bending Moment Acting on the Plate

**Figure 2:**
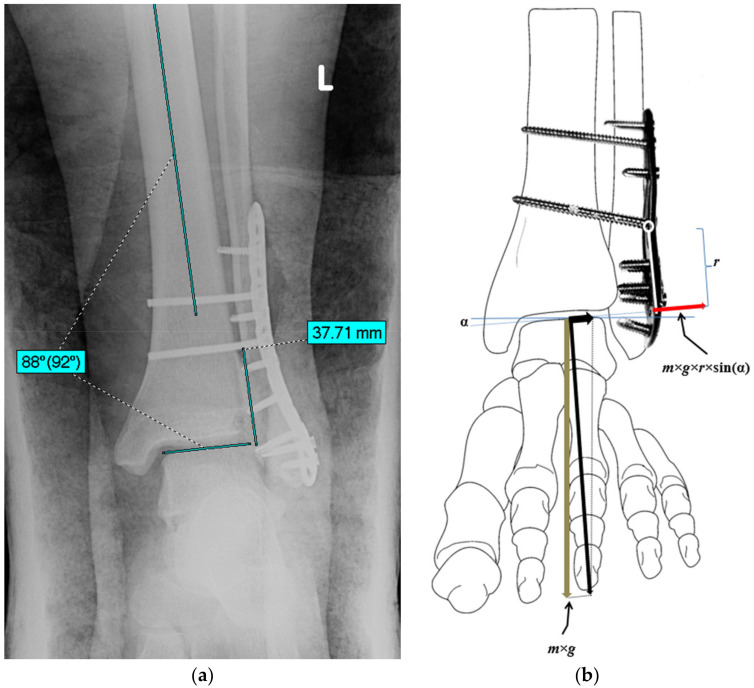
The value of the valgus angle of the ankle joint (α = 2°) and the distance between the distal screw stabilizing tibiofibular syndesmosis—an arm (r = 37.7 mm) of the bending force was measured using the postoperative X-ray (**a**). Schematic representation of the moment of the force (*m*×*g*×*r*×sin(α)) that was responsible for valgus deformation of the plate (**b**).

The IntelliSpace PACS DCX software (Phillips, Eindhoven, The Netherlands) was used in numerical analysis of the X-ray documentation. It enabled to estimate values of the valgus positioning of the ankle joint to the long axis of the tibia (angle of the “slipping” of the talus in relation to the distal tibia end) and the distance from the most distal screw stabilizing DTFS, and finally, the upper edge of the trochlea of the talus that presses the fibula (arm of the bending force), as presented in Figure 2a. Both values were subsequently applied to calculate the value of the force that was responsible for the bending of the plate, and its moment. It should be emphasized that, while standing on one limb, the value of the bending force could be determined as *m*×*g*×sin(α), and its bending moment as *m*×*g*×*r*×sin(α), where *m* denotes the body mass, *g* is the standard gravity, α denotes the angle of the valgus slope of the ankle, and *r* is the radius of the force action line.

### 2.3. Strength Assessment of the Plate

The maximal admissible bending moment of the distal lateral fibular plate (System 5.0, ChM, Lewickie, Poland) was assessed using the plate dimensions and ultimate tensile strength of the TiAl6V4 alloy. It was assumed that the plate was mainly subjected to the quasi-static bending in the *Oxy* plane (Figure 2b). Thus, the stress limit of this material was considered as the highest admissible stress due to the bending moment action, using the following formula: *Q_b_* = 0.64 × *R_m_*, where *R_m_* denotes the ultimate tensile strength for titanium alloy.

**Figure 3:**
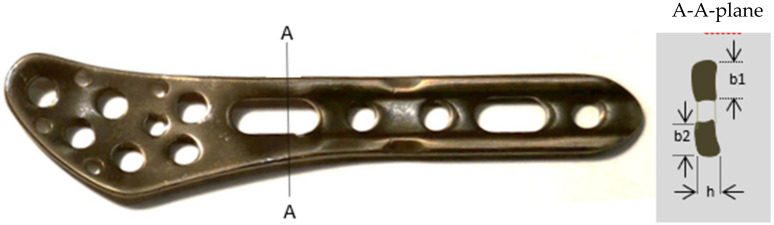
The plate with its most predisposed bending area (A-A) and dimensions of the anterior (*b*_1_ = 4 mm) and posterior (*b*_2_ = 3 mm) branches. For both branches, *h* was the same, equal to 2.3 mm.

The A-A plane (Figure 3) was considered as the most compromised area that undergoes bending based on clinical observations on the one hand, and analysis of the plate shape plus the plate cross-section, where an oval hole occurred, on the other hand. The bending strength index leading to a plastic flow of this cross-section is the sum of two partial indexes: *W* = (*b*_1_ × *h*^2^)/4 + (*b*_2_ × *h*^2^)/4, where *h* is the thickness of the plate in the A-A cross-section, and *b*_1_ and *b*_2_ denote the minimal widths of anterior and posterior branches in this section, respectively. Taking the plate dimensions *h* = 2.3 mm, *b*_1_ = 4 mm, and *b*_2_ = 3 mm, one can obtain *W* = 9.2575 × 10^−9^ m^3^. Thus, the maximal stress could be determined as: $\sigma_{max}=\frac{k_{1}M_{max}}{W},$ where *M_max_* denotes the expected maximal bending moment acting on the ankle, and *k*_1_ is the stress intensity factor taken into consideration due to the possible notch effect at the A-A cross-section [16]. Assuming *σ_max_* = *Q_b_*/*k*_1_, the maximal admissible bending moment could be determined from the equation: $M_{max}=W\frac{\sigma_{max}}{k_{1}}$. The reasonable value of *k*_1_ was estimated as 1.3.

### 2.4. An Analysis of Constitutive Parameters of Patients Treated in the Department of Orthopedic Surgery and Traumatology

To compare observations performed on the patient considered in this paper, a detailed analysis of age, sex, body mass, and height of patients treated in the Department of Orthopedic Surgery and Traumatology, Medical University (Warsaw, Poland), was carried out in the same year, from 1 January to 31 December 2017. Patients under 18 years old and those presenting significant metabolic and genetic disturbances, including Down syndrome, achondroplasia, hypophosphatemia, etc., as well as patients with incomplete data records, were excluded from this research. Finally, a group of 2007 patients was created, consisting of 1115 women and 892 men, aged 18–98 years (55.2 ± 18.5, mean ± SD). The analysis performed in the above-described group of patients showed that an average body mass was equal to 78.1 ± 17.0 kg, with a BMI of 27.2 ± 5.1 kg/m^2^ (mean value ± standard deviation). The patients’ body mass and BMI ranged from 31 to 155 kg and from 12.4 to 57.6 kg/m^2^, respectively (Table 1 and Table 2). It should be highlighted that the percentage of patients with a body mass exceeding 75 kg reached 56%, and for those exceeding 100 kg, 11.7%. This reflects both an increasing number of overweight and obese populations in modern societies and the fact that obese people are much more prone to injuries and disorders of the musculoskeletal system [17,18,19]. Moreover, their treatment is encumbered with a higher complication rate [20]. Without any doubt, obesity increases the risk of destruction of implants stabilizing long bone fractures [21,22] and loss of joint prostheses [16].

## 3. Results

The X-ray observations revealed valgus positioning of the ankle joint to the long axis of the tibia at two degrees and the distance from the most distal screw stabilizing DTFS, as well as an upper edge of the trochlea of the talus of 37.7 mm. Further calculations enabled to determine the bending force and bending moment, acting on the plate, while standing on one limb as 41.9 N and 1.55 Nm, respectively.

The plate’s geometry and TiAl6V4 alloy’s ultimate tensile strength of 1170 MPa allowed to estimate the highest admissible stress due to the bending moment action (*Q_b_*) of 750 MPa.

Thus, the calculated maximal admissible bending moment that could be transmitted by the plate at the A-A section without permanent deformation ($M_{max})$ was equal to 5.34 Nm.

An analysis of constitutive parameters of patients treated in the Department of Orthopedic Surgery and Traumatology revealed that 682 of them (34.0%) had a BMI that could be classified as “optimal” (BMI between 18.5 and 25.0 kg/m^2^), and 38 (1.9%) were underweight, i.e., with a BMI lower than 18.5 kg/m^2^. On the other hand, 756 patients were overweight, i.e., with a BMI between 25 and 30 kg/m^2^, and 531 were obese, i.e., with a BMI greater than 30 kg/m^2^, forming the largest group: *n* = 1287 from 2007 patients (64%) (Table 3).

It should be emphasized that the body mass of all 2007 patients varied from 31 to 155 kg (78.1 ± 17.0 kg), including 722 (36.0%) with a body mass below 70 kg, 363 (18.1%) with a body mass between 70 and 80 kg, and 922 (45.9%) with a body mass above 80 kg. It is worth mentioning that more than half of the patients from the group of 1139 from 2007 patients, i.e., 56.7%, had a body mass larger than 75 kg, including 237 of them (11.8%) with a body mass larger than 100 kg (Table 1).

## 4. Discussion

In the above-described case, deformation of the plate stabilizing the distal end of the fibula led to the gradual lateral displacement of the trochlea between malleoli and elongation of the deltoid ligament. It occurred due to the weight bearing on the limb by a young obese patient. This displacement was caused by two-degrees valgus positioning of the ankle, obesity, and limb loading. These factors generated the resultant force, that was carried by the talus on the lateral ankle and consequently led to the bending of the stabilizing plate. The force, while standing on one leg, was estimated to be equal to 41.9 N, whereas its bending moment was equal to 1.55 Nm.

As a matter of fact, the calculated maximal admissible bending moment that should not deform the plate permanently (5.34 Nm) was 3.5 times higher, and therefore, repetitive loading should not break it. However, our calculations assumed a static case only. In cases of dynamic loading, some overloads that often exceeded the static ones several times could potentially occur [23]. The finite element modeling calculations showed that regular walking generates loads reaching peak values of 3.2 [24], landing from a jump from 2 to 12, and running even up to 13-times higher than the body weight [25,26]. This indicates that the dynamic loading of the plate during regular walking alone could lead to its break, especially in the case of the male with a body mass of 120 kg. The detailed investigations on the effect of dynamic loading on the mechanical response of an implant subjected to significant overload caused by increased body mass could be performed experimentally by using conventional testing machines, as reported in [27,28].

The static moment of the bending force of 0.97 Nm assessed for a 75 kg individual would not exceed the maximal allowable one, even being multiplied by 3.2, that is reaching the maximal peak values, while walking. Loads exceeding the maximal admissible ones should also not be generated if an obese patient were to obey recommendations. Unfortunately, in the analyzed case, the obese patient loaded the extremity intensively while walking.

It should be mentioned that in the late fifties of the XX century, the AO Group (Arbeitsgruppe für Osteosynthesefragen) formulated principles for trauma implants’ construction and application. According to their suggestions, several companies provided implants suitable for skeletal traumatology. These implants met the durability requirements that were applicable those days and were probably established based on the average weight of potential patients.

Nevertheless, during the forthcoming decades, the average human body weight (and height) increased. Thus, higher mechanical loads had to be transduced, and as a consequence, the implants should be much more durable. Application of implants having inadequate strength with respect to a patient’s weight significantly greater than the regular weight may lead to implant overloading, its loosening, or finally, to a premature break.

The problem of overweight and obesity has remarkably increased throughout the world [29], and is also observed in Poland [30]. The case analysis of patients operated on in the Department of Orthopedic Surgery and Traumatology showed that overweight and obesity (BMI greater than 25) were demonstrated by 64% of patients, i.e., 37.6% with a BMI in the range of 25.0–29.9 kg/m^2^ and 26.4% with a BMI greater than 30.0 kg/m^2^ (Table 1). Such information is crucial as implants have been designed for the average body mass of 75 kg, as was authoritative a few decades ago. This could be easily demonstrated by carrying capacities of cars and lifts. It should be stressed that a specific schema of the walking applied by patients who try to mitigate mechanical loads requires a strong constriction of calf muscles. These muscles, acting through the Achilles tendon, compress the ankle joint with an additional force. It was estimated that it may increase the force produced by the weight bearing on regular walking alone by ca. 50% [31]. Dynamic, momentary loads are even higher, as when the foot is fiercely placed on a non-elastic floor it transmits forces that may exceed those originating from the body weight, alone by even three times as much. Thus, regular walking on a flat surface produces dynamic forces that may exceed static ones by even five times as much [32,33].

In the case described above, the implant’s exchange and consecutive limb immobilization for six weeks in a plaster cast enabled to obtain the proper healing. The limb immobilization was applied so that the patient was unable to undertake forbidden activities. The plate was exchanged using exactly the same type, because plates of higher strength were not available at that moment. The plate had to be exchanged because repeated bending jeopardized it. Despite having higher similarity to bone than other alloys in terms of Young’s modulus and durability-to-weight ratio, titanium alloys are still prone to breakage when repeatedly bent due to their high stiffness and low malleability [34].

Plaster casts are nowadays seldom used for immobilization due to several unwanted side effects and poor patient comfort. However, they dominate over orthoses imposing their usage, as they cannot be easily taken off and put on again without visible records. Thus, their usage can still be useful for patients that are less prone to obey recommendations.

The association between obesity and implant complications is widely discussed in the recent literature. Musbahi et al. [35] reported that the revision rate of patients treated with a uni-compartmental knee arthroplasty (UKA) for unexplained pain was the most strongly associated cause of revision in the obese population. Moreover, a trend to increased revision rates with BMI > 30 and > 35 and with fixed bearing devices was observed. Polat et al. [36] suggest that morbid obesity should be treated before surgical planning to extend the implant survival and functional outcomes. Xu et al. [37] concluded that obesity was a significant predictor of poorer improvement in clinical outcome and an increased rate of revision ten years postoperatively of patients who underwent a fixed-bearing UKA. Such observations were also confirmed by Nettrour et al. [38], who found the rate of major component revision surgery to be over five-times greater for morbidly obese patients undergoing medial mobile-bearing UKA than that for patients who were not obese. Parratte et al. [39] stressed that treating musculoskeletal injuries in obese patients is a real challenge for orthopedic surgeons. In every case, the surgeon, patient, and family must be aware of the potential complications and the risk of death, infection, or implant failure resulting from the obesity.

## 5. Conclusions

The results presented in this paper confirmed that implants for lower-extremity fractures may not be durable enough to fit the requirements of overweight and obese patients, since mechanical forces and their moments generated during body weight bearing may exceed the commonly tolerated limit values. To minimize the risk of complications in cases of physically active and insubordinate obese patients, an additional lower-limb immobilization in a plaster cast may be justified. The design of more durable implants for obese and highly active patients should also be taken into consideration in the form of implants’ adaptations to the real requirements.

## Figures and Tables

**Table 1:** The number and the percentage of patients with body mass classified within five categories.

Body Mass (kg)	No. of Patients	%
≤70	722	36.0
70–80	363	18.1
≥80	922	45.9
In total		
≥75 kg	1139	56.7
≥100 kg	237	11.8

**Table 2:** Height, body mass, age, and BMI of patients operated on in the Department of Orthopedic Surgery and Traumatology in a consecutive 12 months (January–December 2017).

BMI (kg/m^2^)	<18.5	18.5–24.9	25.0–29.9	>30	Together
mean	17.4	22.5	27.4	33.9	27.2
SD	1.2	1.8	1.4	3.6	5.2
min	12.4	18.5	25.0	30.0	12.4
max	18.4	24.9	29.9	57.6	57.6
No. of patients	39	682	756	530	2007
%	1.9%	34.0%	37.6%	26.4%	100.0%

**Table 3:** BMI of adult patients operated on in the Department of Orthopedic Surgery and Traumatology.

	Height (cm)	Body Mass (kg)	Age (Years)	BMI (kg/m^2^)
mean	169.1	78.1	55.2	27.2
SD	10.2	17.0	18.5	5.1
min	140	31	18	12.4
max	201	155	98	57.6

## Data Availability

The data presented in this study are available upon request from the corresponding author.
